# Clinical Implications of Metachronous Testicular Tumours

**DOI:** 10.7759/cureus.97079

**Published:** 2025-11-17

**Authors:** Azucena Lirio Armas Alvarez, Angel Alois Osorio Manyari, Irache Abaigar Pedraza

**Affiliations:** 1 Urology, Hospital Don Benito-Villanueva de la Serena, Don Benito, ESP; 2 General Surgery, Hospital Don Benito-Villanueva de la Serena, Don Benito, ESP

**Keywords:** androgen deficiency, bilateral testicular tumours, metachronous testicular tumours, mri of the fistula, orchiectomy

## Abstract

Testicular tumours are the most common solid malignancy in young males. Usually being unilateral and of germ cell tumour type, bilateral testicular tumours are very rare. The postoperative follow-up with imaging studies is of high importance, in order to rule out recurrence or the development of new testicular tumours.

We present the case of a 53-year-old man with a history of left radical orchiectomy and testicular prosthesis placement performed 29 years ago, for testicular teratocarcinoma stage I, who underwent an MRI for the evaluation of a symptomatic anal fistula. MRI of the fistula showed a solid mass in his right testis suggestive of seminoma. The patient had undergone a right radical orchiectomy with an anatomopathological result of seminoma pT1a. CT of the thorax, abdomen, and pelvis and preoperative serum tumour markers were normal. At the four-week postoperative follow-up, the patient reported hot flashes, and his wound showed good appearance. Exogenous testosterone was prescribed, and the patient was referred to an oncologist who decided no adjuvant treatment was necessary. At the one-year follow-up, the patient was asymptomatic. Control CT and serum tumour markers were within normal limits.

Long-term follow-up is necessary in patients with a previous history of testicular tumour, using imaging studies to detect recurrence or a new testicular tumour. Testis-sparing surgery should be considered as the first option when tumours are less than 2 cm. Patients with bilateral orchiectomy should start exogenous testosterone as soon as possible.

## Introduction

Testicular tumours are the most common malignancy in young adult men (aged 15-40 years).

Bilateral testicular tumours occur in 1% of cases. Approximately 20% of these tumours are synchronous, and the remainder are metachronous [1]. Synchronous tumours occur at the time of diagnosis or within the first three months after the diagnosis. Metachronous tumours are developing consecutively after a certain time interval.

The risk factors for the development of contralateral tumour are younger patients, seminoma histology, and the presence of tumour or testicular intraepithelial neoplasia [2]. 

The incidence of bilateral germ cell tumours may increase in the future due to the long-term survival of patients after they receive effective treatment for their first tumour [1]. Therefore, regular follow-ups after unilateral orchiectomy are mandatory, using blood tests and imaging studies such as CT and testicular ultrasound [3].

The prognosis of bilateral testicular tumours is good with a survival rate at 20 years of 96.7% [2]. 

## Case presentation

**Figure 1:**
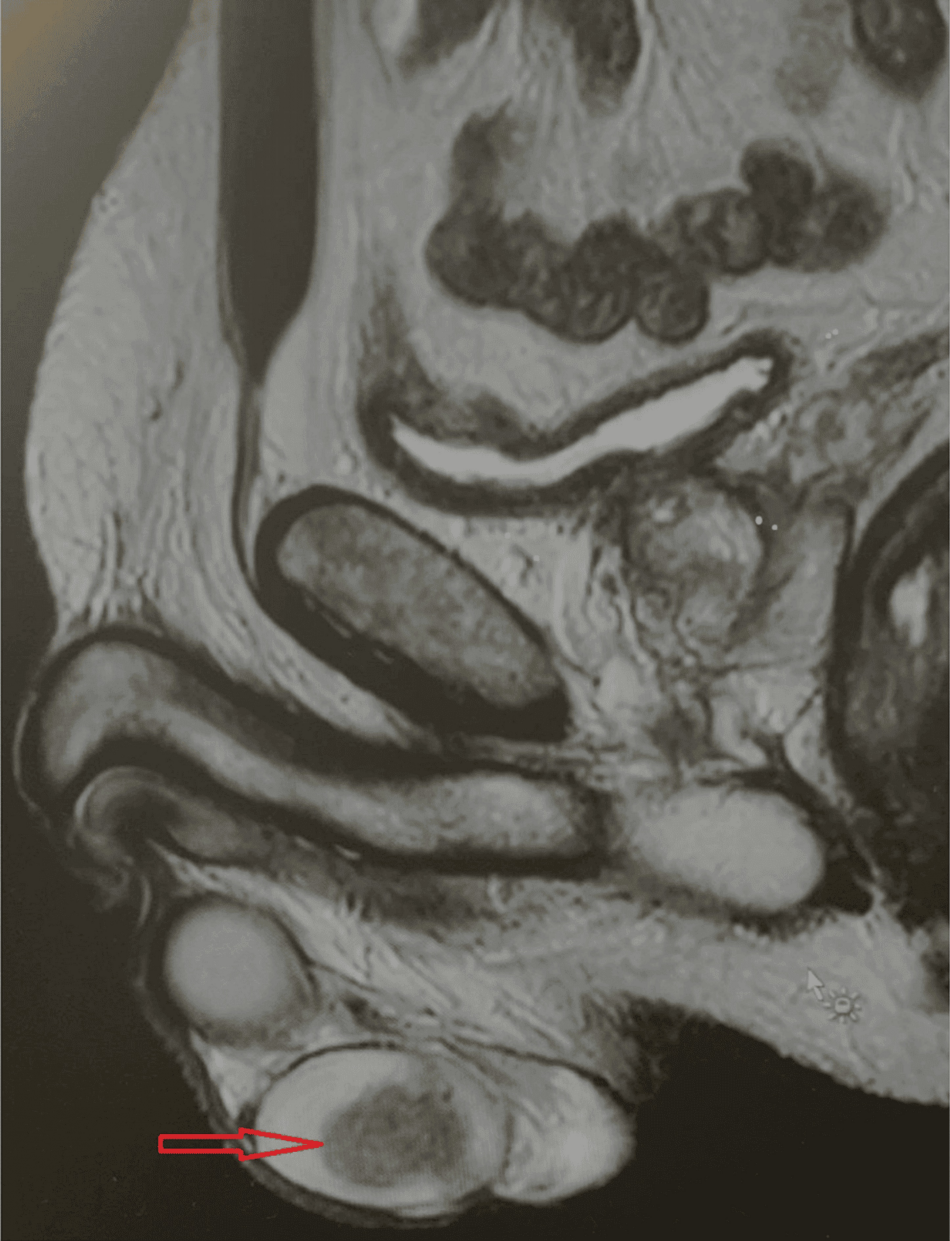
MRI features of seminoma Sagittal T2-weighted image shows an irregular hypointense mass within the right testis causing the distortion of the normal testicular architecture.

A 53-year-old man with a history of left radical orchiectomy and testicular prosthesis placement performed 29 years ago, due to teratocarcinoma stage I, without adjuvant treatment, presented with a symptomatic anal fistula. An MRI of the fistula incidentally showed a 27-mm solid tumour in the right testis suggestive of seminoma (Figure 1). The patient reported no scrotal symptoms, and he did not palpate any masses in his right testis. He underwent right inguinal radical orchiectomy. The immediate postoperative period was uneventful. CT of the thorax, abdomen, and pelvis showed no abnormalities. Preoperative tumour markers were within normal limits: alpha-fetoprotein 1.4 ng/mL (reference range: 0.0-7 ng/mL), beta-human chorionic gonadotropin (β-hCG) 0.2 mIU/mL (reference range: 0.0-2.6 mIU/mL), and lactate dehydrogenase (LDH) 137 IU/L (reference range: 135-250 IU/L).

**Figure 2:**
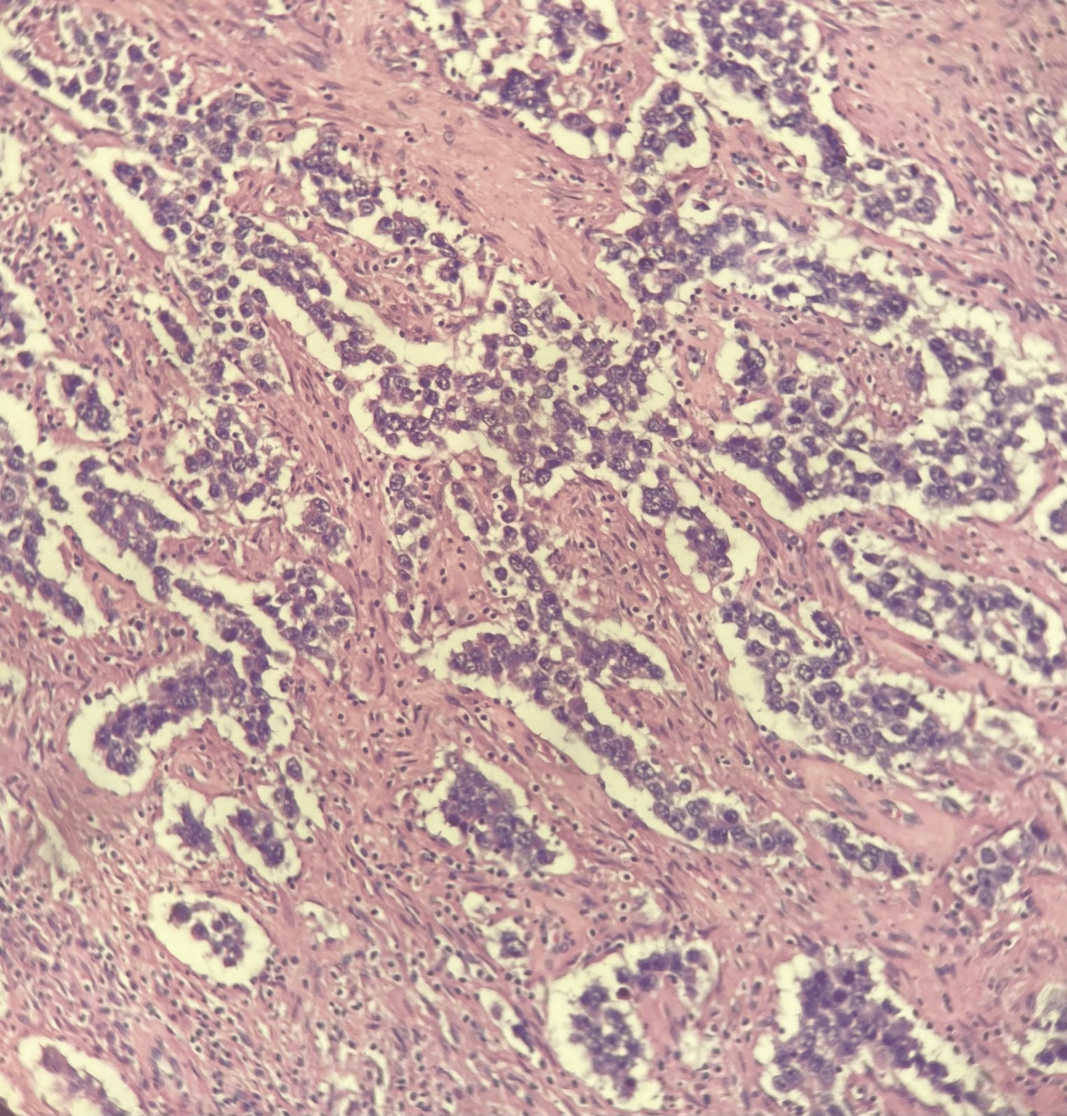
Histological section of seminoma at 20× magnification At 20× magnification, the tumour is composed of well-circumscribed nests and sheets of uniform, polygonal cells with clear cytoplasm and distinct borders, separated by fibrous septa with dense lymphocytic infiltrate. The nuclei are round with prominent nucleoli. No invasion of tunica albuginea, rete testis, or lymphovascular spaces is identified, consistent with stage pT1a seminoma.

The anatomopathological result was seminoma stage pT1a (Figure 2). The tumour was confined to the testicular parenchyma. The epididymis, testicular hilum, and tunica vaginalis were free of neoplasia. No lymphovascular invasion was identified, and surgical margins were free of tumour. Postoperatively, serum tumour markers remained within normal limits: alpha-fetoprotein 1.6 ng/mL, β-hCG 0.2 mIU/mL, and LDH 139 IU/L.

At the four-week follow-up, the patient complained of hot flashes. The surgical wound showed good appearance. Exogenous testosterone was prescribed, and he was referred to an oncologist who opted for surveillance with imaging studies including CT and serum tumour markers rather than adjuvant therapy.

**Figure 3:**
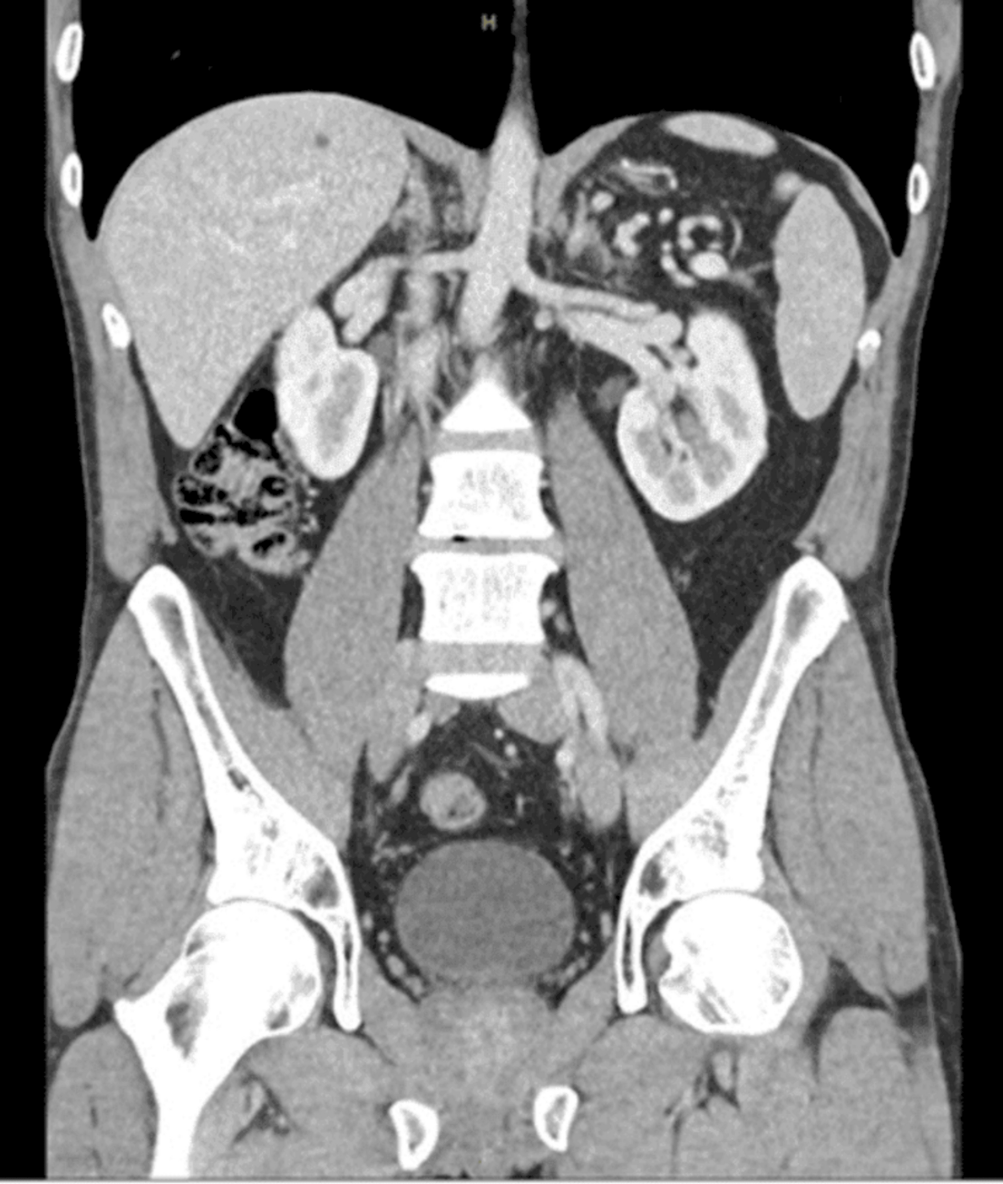
CT of the abdomen and pelvis (coronal view) Contrast-enhanced abdominopelvic CT demonstrates normal findings.

**Figure 4:**
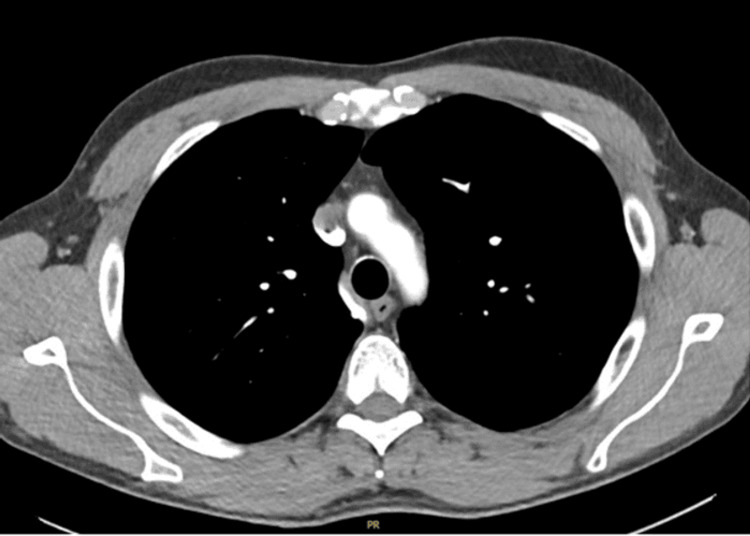
CT of the thorax (axial view) Contrast-enhanced CT of the thorax is unremarkable, with normal lung parenchyma, airways, mediastinum, and pleura.

At the one-year follow-up, the patient was asymptomatic, with normal CT (Figure 3 and Figure 4) and serum tumour markers.

## Discussion

It is very important to follow up on the patients with unilateral testicular tumours who underwent orchiectomy, because there is a 30% risk of recurrence or the development of new tumours in the other testis [3]. Our findings are consistent with those reported in the literature. For instance, a 50-year review identified metachronous testicular tumours occurring after 20 years in six patients (9.5%) and even after 30 years in two patients (3.2%), highlighting the need for long-term follow-up [2].

Follow-ups should be lifelong as new testicular tumours may develop up to 15 years or more after the sentinel event in cases of metachronous bilateral tumour [1]. Imaging studies such as CT and testicular ultrasound and serum tumour markers are the modalities for surveillance in these patients. Additionally, patients with unilateral orchiectomy should be taught to palpate their testes regularly [1]. This is very important because, as in our case, the development of a new testicular tumour may occur asymptomatically, without scrotal symptoms, underscoring the crucial role of imaging studies and self-examination. In contrast, other reported cases have described scrotal manifestations such as testicular pain and tenderness [4].

Testis-sparing surgery should be considered for patients who develop bilateral testicular tumour, metachronous or synchronous, because this type of surgery provides a better quality of life, including factors such as the protection of male body image, fertility, and endocrine function. It may be considered a safe and feasible treatment in tumours less than one-third of the testicular volume or tumours <2 cm [5].

Testicular tumours alter spermatogenesis through mechanisms such as destroying surrounding tissue, the local secretion of human chorionic gonadotropin, elevated intrascrotal temperature, and alterations in local blood flow. Also, the treatment is associated with adverse effects on fertility. So, patients should be informed of the possibility of fertility preservation, including sperm preservation with testicular sperm extraction, if unable to provide semen samples [6-8]. Patients who have undergone bilateral orchiectomy may develop symptoms of androgen deficiency. These symptoms may be physical, sexual, and psychological. Physical symptoms involve weight gain and hot flashes. Sexual symptoms include problems with sexual libido, potency, and ejaculation function. Psychological symptoms are changes in their body image, depression, anxiety, and cognitive dysfunctions [9]. Long-term testosterone supplementation should be considered as soon as possible to avoid these symptoms; additionally, the patients should be monitored regularly with blood testosterone, luteinizing hormone (LH), and follicle-stimulating hormone (FSH) [8]. In our case, the delay in initiating testosterone replacement therapy may explain the patient's symptoms, such as hot flashes. Intramuscular injection of testosterone every two weeks to three months is the most common schedule and is not usually associated with any side effects [8].

In addition, we should inform the patients about lifestyle interventions such as smoking cessation, to reduce the risk of secondary malignancies, plus encourage regular physical activity and a healthy diet. Also, these patients should be assessed by a counsellor and receive psychosocial support [9,10]. 

Genetic testing may have additional relevance for patients with a prior diagnosis of germ cell tumours, who are at higher risk of developing a metachronous contralateral testicular tumour [11-13].

A recent analysis of the Cancer Genome Atlas demonstrated that KIT, KRAS, and NRAS gene variants were observed only in seminoma, while gene variants in B3GNT8, CAPN7, FAT4, GRK1, TACC2, and TRAM1L1 occurred only in embryonal carcinoma [9,14].

Jackson et al., based on their data, suggest that a cellular origin, rather than specific genomic alterations, plays a more critical role in the pathogenesis of bilateral testicular tumours [15].

miRNAs may be used in cancer screening, subtype classification, evaluating treatment response, monitoring residual disease, and predicting disease relapse and as part of surveillance to potentially reduce the frequency of radiological imaging [16-18].

MRI is an efficient diagnostic tool for the evaluation of testicular masses. It is accurate in the preoperative differentiation of benign and malignant intratesticular masses and allows the precise assessment of the local disease extent in patients with malignant tumours [19].

## Conclusions

Long-term follow-up is necessary in patients with previous testicular tumours, using blood tests, CT, and testicular ultrasound to detect recurrence or a new testicular tumour. A high index of suspicion should be maintained for the development of a new testicular tumour in these patients, as such tumours may present asymptomatically. Testis-sparing surgery should be considered as the first option for tumours less than 2 cm. Patients with bilateral orchiectomy should start exogenous testosterone as soon as possible.
